# Serologically Defined Variations in Malaria Endemicity in Pará State, Brazil

**DOI:** 10.1371/journal.pone.0113357

**Published:** 2014-11-24

**Authors:** Maristela G. Cunha, Eliane S. Silva, Nuno Sepúlveda, Sheyla P. T. Costa, Tiago C. Saboia, João F. Guerreiro, Marinete M. Póvoa, Patrick H. Corran, Eleanor Riley, Chris J. Drakeley

**Affiliations:** 1 Instituto de Ciências Biológicas, Universidade Federal do Pará, Belém, Pará, Brazil; 2 Department of Immunology and Infection, London School of Hygiene & Tropical Medicine, London, United Kingdom; 3 Center of Statistics and Applications, University of Lisbon, Lisbon, Portugal; 4 Instituto Evandro Chagas, Ananindeua, Pará, Brazil; Universidade Federal de Minas Gerais, Brazil

## Abstract

**Background:**

Measurement of malaria endemicity is typically based on vector or parasite measures. A complementary approach is the detection of parasite specific IgG antibodies. We determined the antibody levels and seroconversion rates to both *P. vivax* and *P. falciparum* merozoite antigens in individuals living in areas of varying *P. vivax* endemicity in Pará state, Brazilian Amazon region.

**Methodology/Principal Findings:**

The prevalence of antibodies to recombinant antigens from *P. vivax* and *P. falciparum* was determined in 1,330 individuals. Cross sectional surveys were conducted in the north of Brazil in Anajás, Belém, Goianésia do Pará, Jacareacanga, Itaituba, Trairão, all in the Pará state, and Sucuriju, a free-malaria site in the neighboring state Amapá. Seroprevalence to any *P. vivax* antigens (MSP1 or AMA-1) was 52.5%, whereas 24.7% of the individuals were seropositive to any *P. falciparum* antigens (MSP1 or AMA-1). For *P. vivax* antigens, the seroconversion rates (SCR) ranged from 0.005 (Sucuriju) to 0.201 (Goianésia do Pará), and are strongly correlated to the corresponding Annual Parasite Index (API). We detected two sites with distinct characteristics: Goianésia do Pará where seroprevalence curve does not change with age, and Sucuriju where seroprevalence curve is better described by a model with two SCRs compatible with a decrease in force of infection occurred 14 years ago (from 0.069 to 0.005). For *P. falciparum* antigens, current SCR estimates varied from 0.002 (Belém) to 0.018 (Goianésia do Pará). We also detected a putative decrease in disease transmission occurred ∼29 years ago in Anajás, Goianésia do Pará, Itaituba, Jacareacanga, and Trairão.

**Conclusions:**

We observed heterogeneity of serological indices across study sites with different endemicity levels and temporal changes in the force of infection in some of the sites. Our study provides further evidence that serology can be used to measure and monitor transmission of both major species of malaria parasite.

## Introduction

Efforts in mapping malaria transmission have demonstrated a wider geographical distribution of the parasite *Plasmodium vivax* compared to *P. falciparum* [1],[2]. The number of clinical cases due to *P. vivax* infection has been estimated from 106 to 313 million cases per year across the world [3]. In South America, *P. vivax* is currently the most predominant malaria species [2]–[5] and the Brazilian Amazon region has been considered a natural frontier for malaria transmission since 1970 [6],[7], when intense human migration led to a significant increase in the number of malaria cases [8],[9]. In the last two decades, there were reported between 300,000 to 600,000 malaria cases per year in Brazil with *P. vivax* representing 75–80% of these [7].

The control programs have had a significant impact on *P. falciparum* malaria burden in Brazil, which predominated in the past [7]–[9], but only a moderate effect on *P. vivax* infections. The high frequency of asymptomatic *P. vivax* carriers in the Brazilian endemic area [10]–[13] together with a long period of incubation of hypnozoites [14] might be possible explanations for this partial success in controlling this *Plasmodium* species. In this setting of high proportion of asymptomatic carriers, it is critical to have in hand good epidemiological tools in order to assess not only the status quo of disease dynamics, but also to monitor any change in disease transmission due to malaria control interventions.

In Brazil, variations or changes in malaria transmission have been previously associated with intensive use of land and environmental transformations due to farming, deforestation, or gold mining [9],[15]–[17]. Additionally, it has been shown that the proportion of asymptomatic infections in native Amazonian population tends to increase with age [12],[13]. This observation suggests that continuous parasite exposure, even at a low rate, is enough to induce some degree of protective immunity. The combination of these factors implies additional difficulties in assessing the underlying malaria epidemiology of these low transmission and ecologically variable settings.

A large sero-epidemiological study, conducted at the end of the 1960's showed an association between malaria endemicity and the prevalence of antibodies against *Plasmodium* antigens across different South and Central American countries [18]. Later studies using crude or recombinant antigens from *P. falciparum* and/or *P. vivax* have confirmed these findings in Africa [19]–[21], Asia [22],[23] and South America, including Brazil [12] [24]–[30].

Estimation of malaria transmission is routinely based on vector and parasite measures. The possibility of relapses in *P. vivax* infected individuals complicates control significantly and makes statistical inferences over parasite prevalence measures more problematic [2],[31]. Alternatively, serological markers are useful in areas of low endemicity, where it is likely to be easier to detect relatively long-lasting antibody responses than a low prevalence of symptomatic or asymptomatic infections in the host or the entomologic infection rate [19] [32]–[34]. This approach has been applied to the estimation of *P. falciparum* malaria transmission. However, there is a scarce number of studies aiming to test whether seroprevalence for *P. vivax* antigens can be an equally good epidemiological tool to measure and monitor changes in malaria transmission rates. With this goal, we have conducted a cross sectional study where we determined the antibody prevalence to *P. vivax* and *P. falciparum* for individuals living in areas with varying malaria endemicity levels in Pará state, Brazilian Amazon region.

## Methods

### Study area

**Figure 1:**
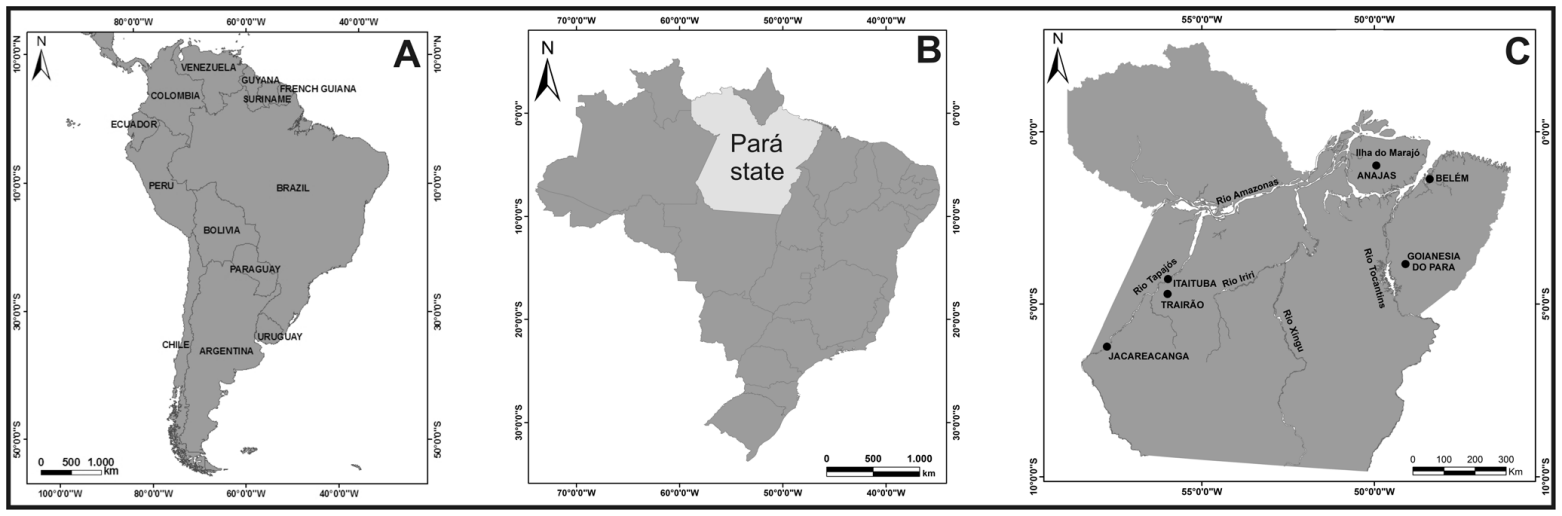
Map of the study area: A. South America, B. Brazil with Pará state in the Amazon region, C. The sampled six municipalities.

The present study was conducted in six municipalities in Pará state in the northern region of the country (Figure 1). Geographically, Pará state is one among eight states that constitute the Brazilian Amazon region and, as such, consists of tropical forest with many rivers. This is an area of intense seasonal rainfall with maximum levels between November and April. Malaria endemicity is typically classified as low endemicity and occurs during and immediately after the rainy season with the peak parasite prevalence typically registered between June and November. The municipalities in this study were selected based on the risk of malaria as estimated by the Brazilian office for malaria surveillance and control [35].

The samples were collected in ten localities within the municipalities considered to be representative of each area. The number varied from one to three localities per municipality. These municipalities and the corresponding localities were: Anajás city (central urban area); Jacareacanga (Porto Rico and São José); Goianésia do Pará (Rouxinol, Santa Paula and Ararandeua); Itaituba (São Luiz do Tapajós); Trairão (Três Boeiras); and Belém city (Cotijuba island). A malaria-free area, Sucuriju village in Amapá state, was also included in the study. The sampling coverage in relation to total residents of each study site was the following: Anajás (12.0%), Belém (15.4%), Jacareacanga (19.3%), Goianésia do Pará (29.3%), Itaituba (48.0%), Sucuriju (61.0%), and Trairão (81.0%). See Table 1 for further details.

**Table 1:** Baseline characteristics of the study area where all municipalities are in the Pará state with the exception of Sucuriju village (in the Amapá state).

Municipality	Year of survey	API^a^	N	Age in years, median (range)	Males, %	Previous exposure, %
Anajás	2009	953.7 (high)	113	29 (2–98)	58.4	89.7
Jacareacanga	2009	138.3 (high)	172	38 (0–75)	43.0	92.4
Goianésia do Pará	2010	198.7 (high)	262	34 (3–71)	45.0	88.1
Itaituba	2007	74.0 (high)	183	23 (1–81)	61.2	44.2
Trairão	2006	54.0 (high)	204	26 (1–82)	54.9	75.7
Sucuriju	2007	4.3 (low)	253	19 (2–83)	50.6	5.0
Belém	2007	0.5 (low)	143	24 (1–80)	49.7	26.6

a Annual Parasite Index at the year of survey with the respective classification (low or high) as reported by the Brazilian Ministry of Health (www.saude.gov.br/sivep_malaria).

Sucuriju village is usually considered as malaria-free area.

### Ethics statement

This study was carried out in full accordance with all International and Brazilian accepted guidelines as written informed consent was obtained from all participants. The Ethics committee reviewed and approved all the consent procedures. A signed consent form was approved for all adults and provided by parents or guardians for children under 15 years old. For individuals between 16 and 18 years old, the consent procedure was similar to adult participants. This study was approved by the Federal University of Pará (3686/2005) and by the Evandro Chagas Institute (0014/2007 and 0031/2010) Ethics committee.

### Samples and data collection

One cross-sectional survey was performed in each study site between 2006 and 2010. The total sample size was 1,330 individuals and ranged from 113 (Anajás) to 262 (Goianésia do Pará) with an average sample size of 190 individuals per municipality. According to the malaria surveillance office of the Brazilian Ministry of Health (SIVEP-Malaria, http://portalweb04.saude.gov.br/sivep_malaria) [35], there are 2 areas (Belém and Sucuriju village) with low Annual Parasite Index (API, number of malaria cases per year/thousand inhabitants) and other 5 (Anajás, Jacareacanga, Goianésia do Pará, Itaituba, and Trairão) with high API at the time of survey (Table 1). Each survey was carried out at meeting points well known by the respective community, such as a health office, a school or a residential house conveniently adapted for medical attendance. Individuals attending these facilities for medical appointments during the survey period were included in the sample. The survey period for each locality was one to two weeks, except in the case of Cotijuba island (Belém), where the sampling were carried out for three months, one survey per week in each month. The samples were collected in the following municipality time periods: Anajás - August 2009, Jacareacanga - February 2009, Goianésia do Pará - November 2010, Itaituba - March 2007, Trairão - October 2006, Belém - between September and November 2007, Sucuriju village in Amapá state - June 2007. For each participant, baseline characteristics were recorded and comprised information on time living in the sampling area and self-reported history of malaria including the number of previous malaria episodes and corresponding symptoms, and, in the case of having reported malaria during the previous year before survey, whether that was confirmed by thick blood smear.

Blood samples were taken by finger prick to prepare thick blood smears and collected into microtainer tubes. The tubes were centrifuged at 2000 rpm with the sera subsequently stored at 4°C. The thick blood smear was examined immediately after and parasitaemia assessed by two experienced microscopists where 200 fields were read from each slide before being declared negative [36].

### Serological assays

All sera were tested for IgG antibodies to recombinant blood-stage *P. vivax* and *P. falciparum* malaria antigens: apical membrane antigen 1 (AMA1) and merozoite surface protein 1 (MSP1) using quantitative enzyme-linked immunosorbent assay (ELISA) as described elsewhere [19],[37],[38]. For *P.vivax* two recombinant proteins of MSP1 (Belém genotype), both contained the 19 KDa C-terminal portion of MSP1 (MSP1_19_) in fusion with histydine tag (His_6_) or Glutationa S-transferase (GST) [37], as well as AMA1 (Salvador genotype) were used. For *P. falciparum* malaria antigens AMA1 (3D7) and MSP1_19_ (Wellcome) [38]. Briefly, Immunolon-4HBX plates were coated overnight at 4°C at a concentration of 0.5 µg/ml of each recombinant antigen diluted in 50 µl per well. Plates were washed using PBS, 0.05% Tween 20 (PBS/T) and blocked using 1% (w/v) skimmed milk powder in PBS/T. After this step, 50 µl of each sample, a positive control (a pool of hyperimmune serum collected from two malaria endemic area, Gambia and Brazil in case of test with antigen from *P. falciparum* and *P. vivax*, respectively) were added in duplicate to each plate. The sera were tested at a final dilution of 1∶1000 for both *P. vivax* recombinant proteins, and 1∶1000 and 1∶2000 for *P. falciparum* MSP1_19_ and AMA1, respectively. The plates were washed and horse-radish peroxidase conjugate rabbit anti-human IgG antibody (DAKO, Roskilde, Denmark) was added to all wells at a dilution 1∶5000 in PBS/T. Antibody responses were detected after development with o-phenylenediamine dihydrochloride substrate solution (OPD) for 20 minutes. Reactions were stopped with 25 µl per well of 2M H_2_SO_4_. Plates were read immediately at 492 nm on a Molecular Devices Versa Max ELISA reader and optical density (OD) values recorded.

### Statistical analysis

Duplicate ELISA OD values were averaged and normalized against the positive control sample on each plate. OD data was then converted to antibody titers, expressed in Arbitrary Units (AU/ml), using a standard curve obtained from an appropriate hyperendemic sera control. Antibody titers were calculated using the formula: titer  =  dilution/[maximum OD/(OD test serum – minimum OD) -1] (33). Seropositivity was determined by fitting a mixture model to normalised OD values. The model assumed two Gaussian distributions, one for sero-negative individuals and another for sero-positive individuals [39]. The mean OD plus three standard deviations associated with the sero-negative group was used as the cut-off value for seropositivity. A separate cut-off was generated for each antigen of each species.

Seroprevalence was stratified into yearly age groups and then analyzed using a reverse catalytic modeling approach under a binomial sampling assumption, as described elsewhere [19],[21],[38],[39]. In this context we aimed to estimate two key parameters (i) the seroconversion rate (SCR or λ) that is the annual rate at which individuals change from seronegative to seropositive and is related to disease transmission intensity [19],[21],[38] and (ii) seroreversion rate (SRR or ρ) that is the annual rate at which seropositive subjects revert to a seronegative state. Each data set was analyzed using two reversible catalytic models, one that assumes a constant-SCR over time and another that assumes a change in SCR at a given time point. The models were estimated by means of maximum likelihood method. In the case of the model assuming a change in SCR, we applied the profile likelihood method to obtain the most likely time point where that change occurred [19],[21]. Where indicated, comparison between these different models was carried out by means of likelihood ratio tests. To obtain confidence intervals and standard errors for SCR and SRR we used bootstrap as follows: (1) re-sample with replacement from the original data, (2) fit the best model to this new data and save the respective estimates, (3) repeat previous steps (e.g., 1000 times) until obtaining a sample of estimates, (4) calculate the standard deviation of the estimates sample associated with each parameter (bootstrap-based standard error) and the 2.5% and 97.5% quantiles (lower and upper bound of the bootstrap-based confidence interval).

To analyze age-adjusted antibody levels, the highest titer level for antigens to the same *Plasmodium* species was calculated for each individual and then log-transformed in order to obtain Gaussian distributions approximately. The corresponding analysis was carried out using the Michaelis-Menten model as available in the ‘vgam’ package for the R software [40]. In the case of *P. falciparum* antibody titers from Jacareacanga, we fitted two Michaelis-Menten curves using the age cut-off obtained from the best catalytic model for the corresponding seroprevalence curve.

Finally, a correlation analysis between pairs of different site-specific measures (e.g., API versus seroconversion rates) was performed using the non-parametric *Spearman's* correlation coefficient. We also used logistic regression to correlate previous malaria exposure with age adjusting for municipality and gender.

All statistical analysis was done in the R software (version 3.0, htpp://cran.r-project.org) using our own scripts.

## Results

### Baseline data reveals regional malaria endemicity variations

The number of individuals recruited at each site ranged from 113 to 262 (Table 1). The percentage of participants ranged from 12% (Anajás) to 81% (Trairão) of the total residents in each locality. The percentage of individuals reporting previous malaria infections varied widely across sites, ranging from 5% (Sucuriju) to 92.4% (Jacareacanga) (Table 1). Although some differences across sites can be highlighted in the age distribution and male/female proportion, data suggest two clusters of municipalities: one comprising 5 municipalities (Anajás, Jacareacanga, Goianésia do Pará, Itaituba and Trairão) with higher levels of previous exposure and another including 2 municipalities (Belém and Sucuriju) where malaria exposure is low. Previous exposure is highly and positively associated with age (OR = 1.54 per 10-year increase, CI 95% = (1.40;1.68), p-value <10^−15^; logistic regression adjusted for gender, age and municipality) and older individuals are more exposed to malaria than younger ones. There was also a strong correlation between the respective Annual Parasite Index (API) as reported by Brazilian Ministry Health and reported malaria exposure (ρ = 0.82, Spearman's correlation coefficient, p = 0.03). Data on previous exposure to malaria would appear to reflect the official statistics of the study area.

Seroprevalence to any *P. vivax* or *P. falciparum* antigens, or to either *Plasmodium* species, also showed variation between municipalities (Table 2). Seroprevalence for any *P.vivax* antigens ranged from 19.6% (Belém) to 77.5% (Goianésia do Pará), while seroprevalence for any *P. falciparum* antigens is estimated to be between 0.8% (Sucuriju) and 59.3% (Jacareacanga). As expected, seroprevalence to any *P. vivax* antigens was higher than that to any *P. falciparum* ones in every study site. Parasite prevalence was low and ranging from 0 to 5%. Official API was highly correlated to the seroprevalence to any *P. vivax* antigen, but only moderately correlated with that to any *P. falciparum* antigen (ρ_API- vivax_ = 0.86, p = 0.02 versus ρ_API-falciparum_ = 0.64, p = 0.14, Spearman's correlation coefficient). This likely reflects the difference between the contributions of *P. vivax* and *P. falciparum* to malaria burden, with API representing all malaria cases.

**Table 2:** Parasite prevalence using thick blood smear and seroprevalence to any *P. vivax* and *P. falciparum* antigens and to any species.

Municipality^a^	Parasite prevalence, %	Seroprevalence, % (95% confidence interval)		
		*P. vivax*	*P. falciparum*	Any species
Anajás	1.8 (0.2–6.2)	68.1 (58.7–76.6)	21.2 (14.1–29.9)	73.5 (64.3–81.3)
Jacareacanga	2.3 (0.6–5.8)	69.2 (61.7–76.0)	59.3 (51.6–66.7)	82.0 (75.4–87.4)
Goianésia do Pará	5.0 (2.7–8.3)	77.5 (71.9–82.4)	45.8 (39.7–52.0)	87.8 (83.2–91.5)
Itaituba	0.0 (0.0–2.0)	37.7 (30.7–45.2)	12.0 (7.7–17.6)	41.5 (34.3–49.0)
Trairão	1.5 (0.3–4.2)	64.2 (57.2–70.8)	27.0 (21.0–33.6)	68.6 (61.8–74.9)
Sucuriju	0.0 (0.0–1.4)	28.1 (22.6–34.0)	0.8 (0.1–2.8)	28.9 (23.4–34.9)
Belém	0.0 (0.0–2.5)	19.6 (13.4–27.0)	2.8 (0.8–7.0)	21.0 (14.6–28.6)
All samples	1.7 (1.0–2.5)	52.5 (49.8–55.2)	24.7 (22.4–27.1)	58.1 (55.4–60.8)

a Municipalities sorted by API at the year of the survey as shown in Table 1.

### Seroconversion rates and antibody levels to any *P. vivax* antigens and characterization of study sites by endemicity level

**Figure 2:**
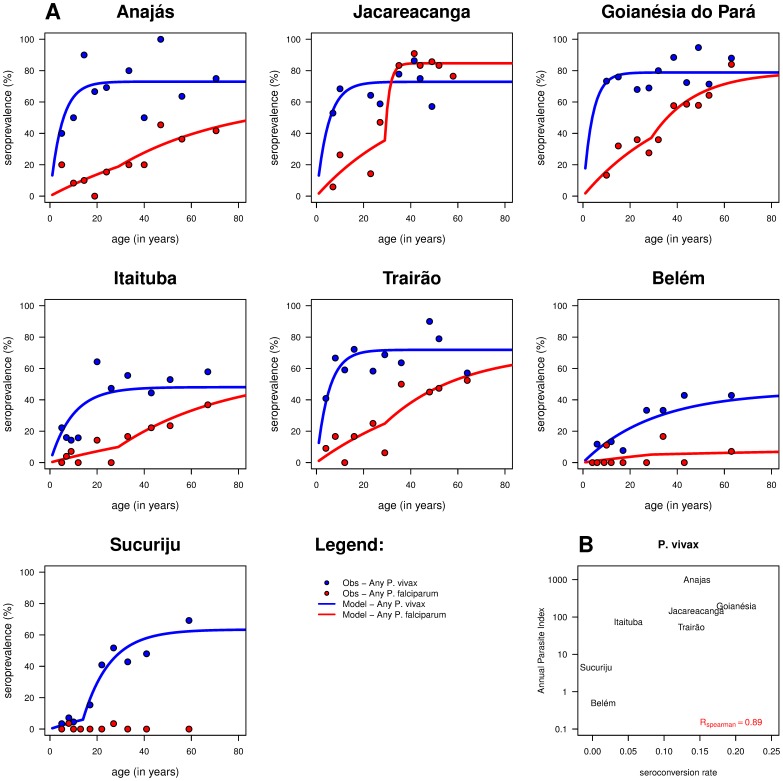
Statistical analysis of seropositivity data. **A**. Age-adjusted seroprevalence for any *P. vivax* (blue solid lines) or *P. falciparum* antigens (red solid lines) using appropriate reversible catalytic models. The observed seroprevalences for each *Plasmodium* species (blue- and red-filled circles) were pooled according to the 10%-centiles of the underlying age distribution. Note that Sucuriju data for *P. falciparum* were excluded from the analysis due to the small number of sero-positive individuals (two cases only). **B.** Correlation analysis for any *P. vivax* antigens using the annual parasite index versus seroconversion rate.

Seroconversion rates (SCR) were estimated in different study sites (Table 3). For *P. vivax* seroprevalence, the best model for the data assumes a stable transmission throughout time but with different transmission intensities across sites with the exception of Sucuriju in the Amapá state (Figure 2A). SCR estimates for disease transmission range from 0.005 (Sucuriju) to 0.201 (Goianésia do Pará), and are strongly correlated to the corresponding yearly API (r_SCR-API_ = 0.89, Spearman's correlation coefficient) (Figure 2B). In the case of Sucuriju where malaria endemicity is low according to the Brazilian Ministry Health [35], the data is better described by a model where a 92.8% drop in SCR (from 0.069 to 0.005) appears to have occurred 14 years before our data collection. SCR estimates for *P. vivax* were not correlated with other measures of transmission intensity, such as parasite prevalence (presumably because of the low numbers of individuals who were found to be parasite positive). All positive individuals were infected with *P. vivax*, except for one case in Goianésia do Pará which was infected with *P. falciparum* (Table 2). There are few entomological inoculation rate (EIR) estimates for *P. vivax* to allow a robust and reliable correlation.

**Table 3:** Statistical analysis of age-adjusted seroprevalence data using reversible catalytic models.

Seroprevalence	Municipality	λ_current_ (95% CI)	λ_past_ (95% CI)
Any *P. vivax*	Anajás	0.146 (0.096,0.236)	-
	Jacareacanga	0.145 (0.096,0.234)	-
	Goianésia do Pará	0.201 (0.125,0.316)	-
	Itaituba	0.050 (0.034,0.072)	-
	Trairão	0.138 (0.092,0.203)	-
	Sucuriju	0.005 (0.001,0.010)	0.069 (0.030,0.220)
	Belém	0.015 (0.009,0.023)	-
Any *P. falciparum*	Anajás	0.008 (0.003,0.013)	0.014 (0.001,0.040)
	Jacareacanga	0.017 (0.009,0.030)	0.514 (0.042,26.272)
	Goianésia do Pará	0.018 (0.012,0.024)	0.047 (0.023,0.075)
	Itaituba	0.004 (0.001,0.007)	0.014 (0.001,0.029)
	Trairão	0.011 (0.006,0.017)	0.024 (0.005,0.058)
	Sucuriju	-	-
	Belém	0.002 (0.000,0.004)	<0.001 (0.000,0.007)

For *P. vivax* seropositivity data the best model assumes (i) a single seroconversion rate for every site apart from Sucuriju where a changed in seroconversion rate seemed to have occurred 14 years ago and (ii) two seroreversion rates, one for Anajás, Jacareacanga, Goianésia do Pará, Itaituba and Trairão (ρ = 0.054, 95%CI = (0.033,0.081)), and another one for Sucuriju and Belém (ρ = 0.018, 95%CI = (0.002,0.039)). For *P. falciparum* data, the best model assumes: (i) a change in force of infection occurred 29 years ago and (ii) a common seroreversion rate for all study sites (ρ = 0.007, 95%CI = (0.001,0.029)). Data of Sucuriju was not analysed due to a low number of seropositive individuals for any *P. falciparum* antigens.

**Figure 3:**
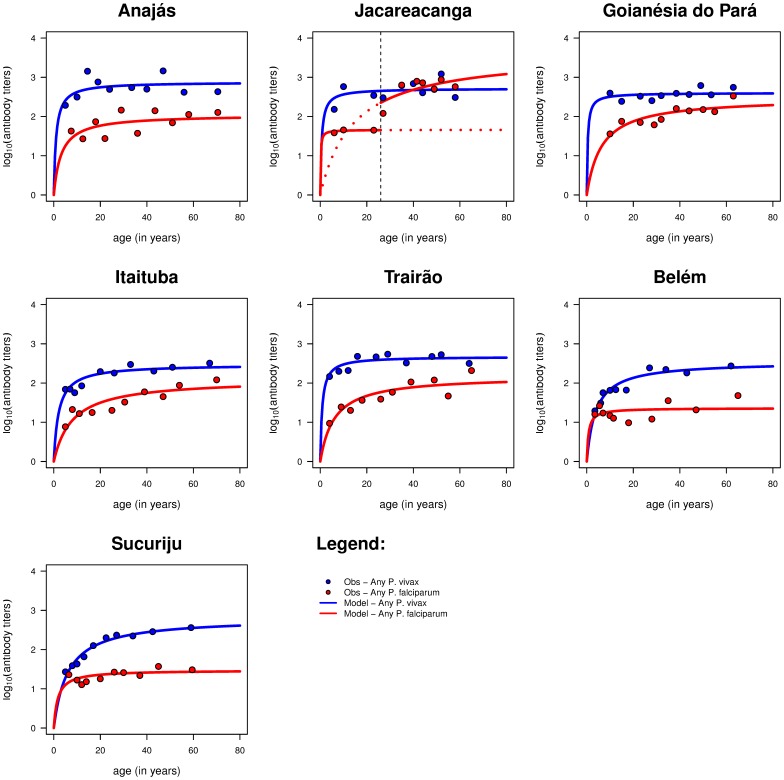
Statistical analysis of antibody titers data. Age-adjusted log10-transformed antibody titer profiles for any *P. vivax* (blue solid lines) or *P. falciparum* antigens (red solid lines) using appropriate Michaelis-Menten models. Blue- and red-filled circles represent the observed mean antibody titer after pooling the data according to the 10%-centiles of the underlying age distribution. The antibody levels refer to the maximum among these reacting to AMA1 and the MSP1 antigens.

Likewise for age-adjusted seroprevalence curves, *P. vivax* antibody titers also increase with age and then reaching a plateau (Figure 3). Variations were observed for this plateau but these do not exceed one order of magnitude across sites. More importantly, variation seems more easily defined by the speed by which the plateau is reached, thus reflecting the rate of malaria exposure. Overall, the sites seem to be divided into two main clusters in line with those defined earlier based on API (Figure S1A). The first one is formed by Anajás, Jacareacanga, Goianésia do Pará and Trairão, where the *P. vivax* antibody level plateau is achieved at a younger age (∼20 years old) due to higher malaria transmission and exposure. In the second cluster - Itaituba, Sucuriju, and Belém - the peak antibody level is reached at an older age (∼30 years old), reflecting a low malaria exposure. Similar clustering can be observed using age-adjusted seroprevalence curves (Figure S1B).

### Comparison of *P. vivax* and *P. falciparum* serological data

All *P. falciparum* seroconversion curves were best described by a model assuming a change in disease transmission occurred ∼29 years before our sampling was conducted (Table 3). Historical SCR (older than 29 years ago) varied between <0.001 (Belém) to 0.514 (Jacareacanga) while current SCR range from 0.002 (Belém) to 0.018 (Goianésia do Pará). No model was fitted to Sucuriju data because of its low seroprevalence (two seropositive individuals only, Table 2). Using SCR-EIR relationship established for *P. falciparum* in Africa, these values represent an overall range of EIR of >0.1 infectious bites per person per year (ib/p/yr) to 1 ib/p/yr except in Jacareacanga where the historical SCR (λ_1_) suggests an EIR of >20ib/p/yr.

In line with the seroprevalence data, the age-adjusted antibody profiles are lower in the *P. falciparum* than in *P. vivax* antibody data (Figure 3). These profiles show little variation at younger ages but a wide range of plateaus older ages (Figure S1B). In the case of Jacareacanga, we could detect a change point in the age-adjusted antibody titer curve around 26 years old similar to that of the corresponding seroprevalence curve.

## Discussion

This study reports data on antibodies responses to AMA-1 and MSP1_19_ antigens from *P. vivax* and *P. falciparum* in 1,330 individuals living in different malaria transmission areas in the Pará state, Brazil. Specific IgG antibody responses, either as titer or seroprevalence, are in good agreement with government-derived API estimates. For *P. vivax*, the minimum and maximum SCRs were obtained in Belém and Goianésia do Pará with the lowest and the highest number of reported malaria cases, respectively. For each municipality, age-adjusted seroprevalence curves for *P. vivax* antigens were higher than the ones for *P. falciparum* antigens, reflecting that the predominance of infections of that species in the different study sites. In agreement with this observation, antibody titers to *P. vivax* antigens tend to reach their corresponding plateau sooner than its *P. falciparum* counterparts. These data support previous work in areas where these species are co-endemic [41],[42], showing that SCR provides a reliable tool for assessing malaria endemicity for both species.

For the serological analysis of *P. vivax* reponses, age-adjusted seroprevalence curves are best fit by simple catalytic models that assumes a stable SCR throughout time but different estimates for the sites. These differences in SCR estimates are likely to reflect the different ecological factors that affect malaria exposure and the acquisition of immunity to malaria in the area. For example, gold mining is the predominant activity in the riverine Jacareacanga. It was previously shown that mining populations exhibit acquisition of immunity to malaria [43]. Mining can also contribute to an increase in number of mosquito breeding sites due to extensive borrow pits dug along mining sites, mimicking the riverine floodwater impoundments [9]. The deforestation occurred in Goianésia do Pará might have been an important factor to the increased malaria risk in the municipality, as observed elsewhere [17]. The coastal city of Belém, as the state capital, is instead more likely to be affected by imported malaria cases due to migration from the surrounding areas. An outlying site is Sucuriju in the Amapá state, where a model assuming distinct disease exposures between younger (<15 years olds) and older individuals (≧15 years old) provides a better fit to the data. Since this site has had no recorded malaria cases in recent years, possible explanation is a past migratory wave to Sucuriju from other parts of Amapá or from another surrounding state with ongoing malaria transmission. Migration is likely to be age and season specific as reviewed elsewhere [9] [44] but the exact nature needs further investigation.

For *P. falciparum*, data suggests a change in transmission occurred ∼29 years before our data collection. This estimate for the time of change in transmission is in line with the extensive wave of emigrants from different parts of Brazil to sites surrounding mining activities occurred in 1980s [9]. As a reaction of migration to mining and farming sites, the Brazilian health authorities initiated several malaria control programs. One of these programs was the SUCAM (Superintendencia de Campanhas de Saúde Pública) which, between 1980s and 1990s, used DDT spraying inside dwellings and actively sought and treated of cases using different anti-malarial drugs [9]. Whilst not wholly successful, the implementation of the program probably had a non-negligible impact on the Amazonian circulating parasite population as shown in a recent retrospective study that suggests the occurrence of bottlenecks on the *P. falciparum* parasite population of Itaituba between the 1980s and 1990s [45]. The results further support the potential of this serological approach to describe malaria transmission dynamics in low transmission settings. This has been used for *P. falciparum* before and recently estimated seroconversion rate has been validated with longitudinal data [46]. However, data on *P. vivax* are more limited and this is the first time that seroconversion rates have been generated for malaria-exposed populations in the Amazon region.

There are several potential limitations of this study. The most obvious one concerns the choice and the number of antigens used to evaluate antibody responses. We only used antibodies to two recombinant antigens of each *Plasmodium* species and, therefore, assuming some level of differential immune responsiveness at the individual level, the data might be an under estimate of exposure to infection occurring in the different study sites. Antibodies to other *P. vivax* and *P. falciparum* antigens are available but their role in explaining the natural variation in malaria immunogenicity is still not fully understood. Newer studies have gone some way to identifying antigens or combinations of antigens that may be more robust for this purpose [47],[48].

Other limitations are related to the timing, type and intensity of the sampling approach. In this study data collection was performed over a 5 year period for the different sites. Whilst the different transmission estimates can be compared and contrasted, temporal comparison between sites reflecting changes in malaria transmission over time or with age is more complicated. However, the profile likelihood approach to estimate changes in transmission identifies a range of years/age over which transmission has changed. In this case, the estimated standard deviations were low and, therefore, broad estimate of 29 years for a change in *P.falciparum* is credible. Perhaps, of more concern for the estimates is the relatively low number of individuals sampled in each site. Besides affecting estimation precision, the small sample size limits the likelihood of identifying statistical significant changes in disease transmission over time from reverse catalytic models. The current sample size has allowed estimates change in transmission for *P.falciparum* that is broadly consistent across sites with plausible biological explanations, however this may not be the case for *P.vivax*. Further, and ideally larger, surveys are required to examine if changes in transmission have occurred for *P.vivax* and, if not, one may ask if this is genuinely more stable transmission perhaps as an immunological consequence of hypnozoite infection. Future surveys will almost certainly require larger sample sizes to demonstrate statistically significant changes in transmission when compared to current surveys (Sepúlveda et al. in preparation). Our surveys were also based on a convenience sampling approach rather than structured community based sample. This could have led to bias in those captured by the survey due to proximity of sampling location and/or health seeking behaviour when the survey was conducted at health facilities. We have previously conducted surveys for serological analysis using communal meeting points [19] and also used health facility attendees [34],[49] both approaches providing credible data. In this study for pragmatic reasons, different sampling locations were used at the different sites following recommendations from local contacts. There appears to be no systematic effect of the sampling location on serological data, though we cannot rule this out completely. A standardized approach, such as school surveys [50], would be much more attractive but these are not always operationally or logistically possible.

In summary, we have used serological data from anti-malaria antibody responses to can describe areas of different malaria endemicity in Pará state, Brazil. We have demonstrated that seroprevalence data shows good correlation with government-derived API and is able to detect changes in malaria transmission in the study sites.

## Supporting Information

Figure S1 **Theoretical predictions for the relationship between age, mean antibody titers, and seroprevalance.** **A**. Expected relationships between age and antibody titers for any *P. vivax* antigens using a Michaelis-Menten modelling approach. **B**. Expected relationships between age and seroprevalence for any *P. vivax* antigens using appropriate reversible catalytic models (see Table 3 for the corresponding parameter estimates).
(TIFF)
