# Correction: Serologically Defined Variations in Malaria Endemicity in Pará State, Brazil

**DOI:** 10.1371/journal.pone.0260513

**Published:** 2021-11-19

**Authors:** Maristela G. Cunha, Eliane S. Silva, Nuno Sepúlveda, Sheyla P. T. Costa, Tiago C. Saboia, João F. Guerreiro, Marinete M. Póvoa, Patrick H. Corran, Eleanor Riley, Chris J. Drakeley

The Data Availability statement for this article is incorrect. The correct statement is: The authors confirm that all data underlying the findings are fully available without restriction. All relevant data are within the paper and its Supporting information files.

S1 Dataset is omitted from the list of Supporting Information. It can be viewed below.

## Supporting information

S1 Dataset Supplementary dataset. Epidemiological and serological data set (Cunha et al. 2014, PLOS ONE).
(XLSX)

## References

[1] Cunha MG, Silva ES, Sepúlveda N, Costa SPT, Saboia TC, Guerreiro JF, et al. (2014) Serologically Defined Variations in Malaria Endemicity in Pará State, Brazil. PLoS ONE 9(11): e113357. 10.1371/journal.pone.0113357 25419900 PMC4242530

