# Supplementary figures and images for: Serologically Defined Variations in Malaria Endemicity in Pará State, Brazil
Source: PLoS One. 2014 Nov 24;9(11):e113357. doi: 10.1371/journal.pone.0113357 (PMC4242530; doi:10.1371/journal.pone.0113357)

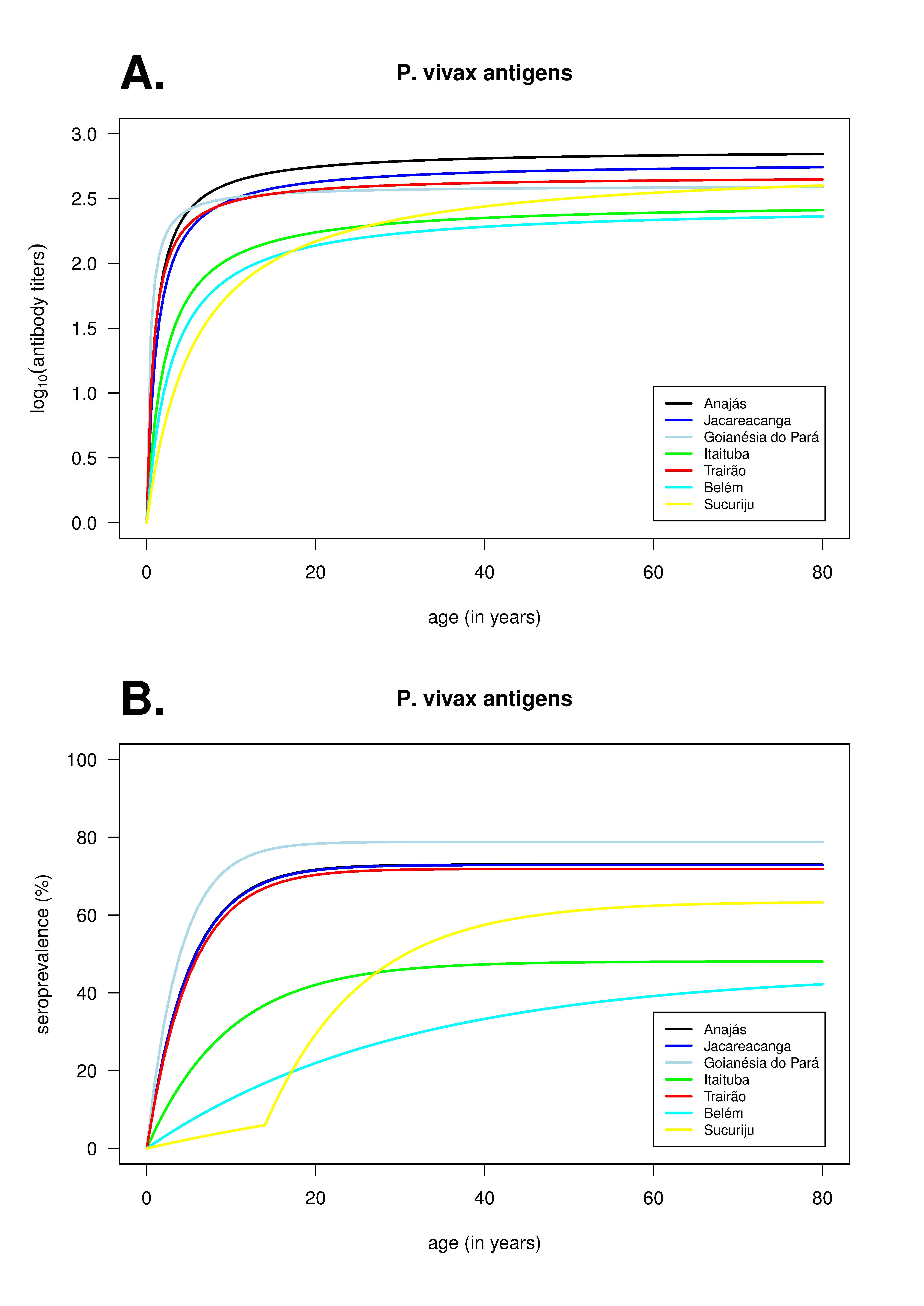

Supplement: Figure S1 — Theoretical predictions for the relationship between age, mean antibody titers, and seroprevalance. A. Expected relationships between age and antibody titers for any P. vivax antigens using a Michaelis-Menten modelling approach. B. Expected relationships between age and seroprevalence for any P. vivax antigens using appropriate reversible catalytic models (see Table 3 for the corresponding parameter estimates). (TIFF) [file pone.0113357.s001.tiff]
